# A newly identified 45‐kDa JAK2 variant with an altered kinase domain structure represents a novel mode of JAK2 kinase inhibitor resistance

**DOI:** 10.1002/1878-0261.13566

**Published:** 2023-12-20

**Authors:** Sivahari Prasad Gorantla, Tony Andreas Mueller, Corinna Albers‐Leischner, Martina Rudelius, Nikolas von Bubnoff, Justus Duyster

**Affiliations:** ^1^ Department of Hematology and Oncology, Medical Center University of Schleswig‐Holstein Lübeck Germany; ^2^ Department of Internal Medicine I University Medical Center Freiburg Germany; ^3^ Department of Internal Medicine I, Center for Molecular Medicine Cologne (CMMC) University of Cologne Germany; ^4^ Department of Oncology, Hematology and Bone Marrow Transplantation with Section Pneumology, Hubertus Wald Comprehensive Cancer Center Hamburg University Medical Center Hamburg‐Eppendorf Germany; ^5^ Department of Pathology University Hospital, LMU Munich Germany

**Keywords:** JAK2‐V617F, myeloproliferative neoplasm, ruxolitinib resistance

## Abstract

Tyrosine‐protein kinase (janus kinase; JAK)–signal transducer and activator of transcription (STAT) signaling plays a pivotal role in the development of myeloproliferative neoplasms (MPNs). Treatment with the potent JAK1/JAK2‐specific inhibitor, ruxolitinib, significantly reduces tumor burden; however, ruxolitinib treatment does not fully eradicate the malignant clone. As the molecular basis for the disease persistence is not well understood, we set out to gain new insights by generating ruxolitinib‐resistant cell lines. Surprisingly, these cells harbor a 45 kDa JAK2 variant (FERM‐JAK2) consisting of the N‐terminal FERM domain directly fused to the C‐terminal kinase domain in 80% of sublines resistant to ruxolitinib. At the molecular level, FERM‐JAK2 is able to directly bind and activate STAT5 in the absence of cytokine receptors. Furthermore, phosphorylation of activation‐loop tyrosines is dispensable for FERM‐JAK2‐mediated STAT5 activation and cellular transformation, in contrast to JAK2‐V617F. As a result, FERM‐JAK2 is highly resistant to several ATP‐competitive JAK2 inhibitors, whereas it is particularly sensitive to HSP90 inhibition. A murine model of FERM‐JAK2 leukemogenesis showed an accelerated MPN phenotype with pronounced splenomegaly. Notably, most current protocols for the monitoring of emerging JAK variants are unable to detect FERM‐JAK2, highlighting the urgent need for implementing next‐generation sequencing approaches in MPN patients receiving ruxolitinib.

## Graphical abstract

Ba/F3 cells expressing JAK2‐V617F treated with 4 μm of ruxolitinib displayed a 45‐kDa JAK2 variant by in‐frame fusion consisting of the N‐terminal 77 amino acids (a.a.) together with residues 814–1132 a.a of the kinase domain (FERM‐JAK2). FERM‐JAK2 is constitutively dimerized and activates STAT5 without receptor interaction. FERM‐JAK2 is resistant toward JAK2‐ATP competitive inhibitors and induces the lethal MPN‐like phenotype in mice.

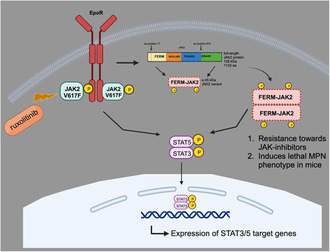

**Abbreviations**
ABL: Abelson murine leukemia
CML: chronic myeloid leukemia
ERK: extracellular signal‐regulated kinase
ET: essential thrombocythemia
GIST: gastrointestinal stromal tumors
HSP90: heat shock protein 90
JAK: janus kinase
JH: JAK homology
MPL: myeloproliferative leukemia
MPN: myeloproliferative neoplasm
NGS: next‐generation sequencing
PCM1: pericentriolar material 1
PMF: primary myelofibrosis
PV: polycythemia vera
STAT: signal transducer and activator of transcription
TEL: translocation‐Ets‐leukemia
TKI: tyrosine kinase inhibitors
WES: whole exome sequencing

## 1 Introduction

The cytoplasmic tyrosine kinase JAK2 plays a major role in the normal development of hematopoiesis and cytokine mediated signaling [1, 2]. Occurrence of a somatic activating mutation valine to phenylalanine (V617F) in the pseudokinase (JH2) domain of JAK2 has been implicated in myeloproliferative neoplasms including polycythemia vera (PV, 90%), essential thrombocythemia (ET, 50%) and primary myelofibrosis (PMF, 50%) [3, 4, 5, 6]. In addition to MPNs, the JAK2‐V617F mutation has also been observed at very low frequencies in the myelodysplastic syndrome, chronic myelomonocytic leukemia (3–8%) and very rarely in systemic mastocytosis [7, 8]. Apart from V617F, several other mutations located in the JH2 domain are also reported in hematological malignancies with very low frequencies such as D620E in a PV patient [9], C661Y in an unclassified MPN [10], L611S in acute leukemic leukemia (ALL) [11], or deletion of an IREED peptide in down syndrome [12]. Subsets of PV patients negative to the JAK2‐V617F mutation showed gain of function mutations affecting JAK2 exon 12 [13]. In addition to the point mutations, JAK2 is also involved as partner in several fusion proteins such as TEL‐JAK2 and PCM‐JAK2 in other hematological malignancies [14]. Biochemical studies have demonstrated that all these mutations lead to constitutive activation of the signal transducer and activator 5 (STAT5) pathway downstream of JAK2. These discoveries encouraged the development of small molecular inhibitors against JAK2, several of which displayed remarkable activity in MPNs, such as ruxolitinib, fedratinib, and lestaurtinib [15, 16, 17]. Consequently, ruxolitinib has been approved by the FDA for the treatment of MPN patients since 2014.

In other oncogenic kinase‐driven diseases such as chronic myeloid leukemia (CML), non‐small cell lung cancer (NSCLC), and gastrointestinal stromal tumors (GIST), acquired resistance to specific kinase inhibitors has been connected to emergence of secondary resistance mutations in the target kinase [18, 19, 20]. However, no ruxolitinib‐resistant JAK2 mutations have been reported so far in MPN patients. In order to predict the drug resistance mechanisms against ruxolitinib, we implemented an *in vitro* screening strategy using leukemic cell lines. Our approach revealed a 45 kDa JAK2 variant (FERM‐JAK2) that confers resistance across a panel of JAK2 inhibitors. Furthermore, a murine mouse model expressing FERM‐JAK2 displayed an accelerated MPN phenotype.

## 2 Materials and methods

### 2.1 Cell culture

Ba/F3 cells (CVCL‐0161) were obtained from the German Resource Centre for Biological Material (DSMZ). Ba/F3 cells were maintained in RPMI 1640 (Gibco, Billings, MT, USA) medium containing 10% fetal calf serum in the presence of 2 ng·mL^−1^ murine IL‐3. These cells were transfected by retroviral gene transfer and transformed upon withdrawal of IL‐3. Phoenix E helper‐virus free ecotropic packaging cells (CVCL‐H717) (a kind gift from G. Nolan, Stanford, USA), HEK293T (CVCL‐0063) and Gamma2A cells (CVCL‐C0D4) (a kind gift from Harvey Lodish, MIT, USA) and NIH3T3 cells (CVCL‐0594) were maintained in DMEM (Gibco) supplemented with 10% FCS. All the cell lines were tested and confirmed mycoplasma free.

### 2.2 Cell line authentication

All cell lines mentioned above were recently authenticated using short tandem repeat (STR) analysis. Validation was performed by Microsynth GmbH in Göttingen, Germany. Profiles are available upon request.

### 2.3 Immunoblotting, co‐immunoprecipitation and *in‐vitro* binding studies

Cell lysis, sodium dodecyl sulfate–polyacrylamide gel electrophoresis (SDS/PAGE), and immunoblotting were done as described previously [21]. Bands were visualized using the enhanced chemoluminescence (ECL) system (Amersham, Braunschweig, Germany). For *in vitro* binding studies both FERM‐JAK2 and JAK2‐V617F were cloned into pcDNA 3.1(+) using the EcoR1 restriction site. Purified STAT5 protein was incubated with *in vitro* translated FERM‐JAK2 or JAK2‐V617F proteins for 3 h. The beads were then washed and bound fractions were subjected to SDS/PAGE and transferred to polyvinylidene difluoride (PVDF) membranes. Bound JAK2 protein was visualized by immunoblotting using anti‐JAK2 antibody.

### 2.4 Sequencing and cloning

Human WT JAK2 and JAK2‐V617F were cloned into EcoRI site of the MiG‐RI retroviral vector expressing the enhanced yellow fluorescent protein (eGFP) as described previously [22]. JAK2 mutations were introduced in MSCV‐EYFP‐JAK2‐V617F using the QuickChange mutagenesis kit (Stratgene, Amsterdam, The Netherlands). Myc‐tagged FERM‐JAK2 was generated by cloning the cDNA into the EcoR1 site of pCMV‐Myc tag vector.

### 2.5 Generation of drug‐resistant variants

Selection of ruxolitinib‐resistant clones was described previously [23]. Briefly, Ba/F3 MSCV‐EYFP‐JAK2‐V617F cells were cultured in 96–well plates at a density of 4 × 10^5^ cells per well in the presence of ruxolitinib at indicated concentrations. Colonies that became visible after 14–20 days were picked, expanded, and analyzed. Resulting inhibitor‐resistant sublines were cultured in the presence of inhibitor at a concentration corresponding to that used during the screen.

### 2.6 Inhibitors and cytokines

Ruxolitinib (INCB018424) was a kind gift from Novartis Pharma AG, Basel, Switzerland. Fedratinib (TG101348) was purchased from Selleckchem (Houston, TX, USA). JAK inhibitor‐1 (JAK inh‐1.) was purchased from Calbiochem (San Diego, CA, USA). 17‐AAG and Geldanamycin were purchased from Sigma‐Aldrich (Taufkirchen, Germany). All inhibitors were dissolved in dimethyl sulfoxide (DMSO) to prepare stock solutions of 10 mm and were stored at −20 °C. Murine interleukin 3 (IL‐3) and human erythropoietin (Epo) were purchased from R&D Systems (Wiesbaden, Germany) and a used in 20 ng·mL^−1^ concentration for stimulation experiments.

### 2.7 Antibodies

The anti‐phosphotyrosine antibodies PY20 and 4G10 were purchased from BD Biosciences (Heidelberg, Germany) and Upstate Biotechnology (Lake Placid, NY, USA). STAT5 (G‐2), pJAK2 (21870‐R), IL‐3Rβ chain (K‐17), Tubulin, heat shock protein HSP90 and HA‐tag (F‐7) antibodies were obtained from Santa Cruz Biotechnology (Heidelberg, Germany). JAK2 c‐terminal antibody (D2E12 XP^R^), pSTAT5, pAKT, pERK, ERK, AKT were purchased from Cell Signaling Technologies (Leiden, The Netherlands). A JAK2 antibody recognizing the pseudokinase domain against the amino acids 750–757 was purchased from Upstate Biotechnology. The anti‐FLAG antibody was purchased from Sigma (Taufkirchen, Germany) and anti‐Myc‐tag antibody from Biozol (Eching, Germany).

### 2.8 Proliferation assay

Proliferation was measured using an MTS (3‐(4,5 dimethylthiazol‐2‐yl)‐5‐(3‐carboxymethoxyphenyl)‐2‐(4‐sulfophenyl)‐2H‐tetrazolium)‐based method by absorption of formazan at 490 nm (CellTiter 96; Promega, Madison, WI, USA). Measures were taken as triplicates after 72 and 96 h of culture without cytokines as described previously [22].

### 2.9 Sequencing and primers

Total RNA was extracted with TRIzol reagent (Invitrogen, Carlsbad, CA, USA). For RT‐PCR of JAK2 encompassing the kinase domain, the following primers were used: JAK2 RT–KD forward 5′‐gaaaatgacatgttaccaaatatg‐3′ and JAK2 RT‐KD reverse 5′‐ggagtaaacaaactgttaaag‐3′. Sequencing was performed by Eurofins Genomics, Ebersbach, Germany. For kinase domain sequencing the following primers were used: Forward 5′‐ctagggttttctggtgcctttgaag‐3′ and reverse 5′‐gggcgttgatttacattattgttcc‐3′. For sequencing of the FERM‐JAK2 the following primers were used: Forward 5′‐atggcctgccttacgatgacagaaatg‐3′ and reversed 5′‐tcttggtaatcttccattattcttcaaaa‐3′. Cloning of the p45 kDa of JAK2 was performed using following primers: Forward 5′‐gatcgaattcatggattacaaggatgacgacgat‐3′ and reverse 5′‐gatcgaattctcatccagccatgttatcccttatttg‐3′. Primers used for site‐directed mutagenesis of phospho‐deficient mutants will be provided upon request.

### 2.10 Bone marrow transduction and transplantation

Bone marrow transduction and transplantation was done as described previously [24]. Briefly, Phoenix E cells were transiently transfected using Lipofectamine 2000 (Invitrogen, Karlsruhe, Germany) and retroviral stocks were collected twice at 12‐h intervals beginning 24 h after transfection. Retroviral titers were determined by transduction of 5 × 10^4^ NIH3T3 cells with serial dilutions of retrovirus in the presence of 4 μg·mL^−1^ polybrene (Sigma). Forty‐eight hours post transduction, the percentage of infected cells was determined by flow cytometric analysis of eGFP‐expression. The titer (in Colony Forming units per ml [CFU·mL^−1^]) was calculated by multiplication of the total number of eGFP‐positive cells with the dilution factor of the retroviral supernatant. Murine bone marrow (BM) was harvested from male Balb/C donor mice 4 days after injection of 150 mg·kg^−1^ 5‐fluorouracil (Ribosepharm, Munich, Germany) and stimulated overnight in Iscove modified Dulbecco medium (Gibco) with 20% FCS supplemented with growth factors (10 ng·mL^−1^ mIL‐3, 10 ng·mL^−1^ mIL‐6, 50 ng·mL^−1^ mSCF, R&D Systems). Primary murine BM cells were transduced by spin infection (1200 ***g***, 32 °C, 90 min) using retroviral supernatant supplemented with growth factors and 4 μg·mL^−1^ polybrene (Sigma). Subsequently, cells were resuspended in Hanks balanced salt solution (Sigma) and injected into the tail vein of lethally irradiated (800 rad) female Balb/C recipient mice. Animals that received a transplant were monitored for signs of disease by serial measurement of peripheral blood (PB) counts. All animals were caged in a special caging system (Thoren Caging Systems, Hazleton, PA, USA) with autoclaved food and acidified water. Balb/C mice was purchased from Charles River (Sulzfeld, Germany). All mice were maintained in a specific pathogen‐free environment and were used between 6 and 8 weeks of age. All procedures were reviewed and approved by the animal welfare officers of the Technical University of Munich and the animal ethics committee of the local government Munich under the license number (Az:55.2‐1‐54‐2531‐75‐08v).

### 2.11 Analysis of transplanted mice

Two independent transplantation experiments were performed, including a total number of at least 15 mice per group. Hemoglobin (Hb), hematocrit (HCT), platelets, and white blood cell counts (WBC) were determined using an automated counter (SCIL vet abc, Heska, Fort Collins, CO, USA). Reticulocytes were stained with brilliant cresyl blue solution (1%) and quantified by light microscopy (per 1000 erythrocytes). Numbers of transduced eGFP‐positive cells in the peripheral blood of transplanted mice were determined by flow cytometric analysis. Spleen and BM specimen were fixed in buffered formalin (4% pH 7.4), decalcified in EDTA and stained with hematoxylin/eosin and Gomori's reticulin staining prior to microscopy.

## 3 Results

### 3.1 Ruxolitinib‐resistant clones display a truncated form of JAK2 with a molecular weight of 45 kDa

**Fig. 1:**
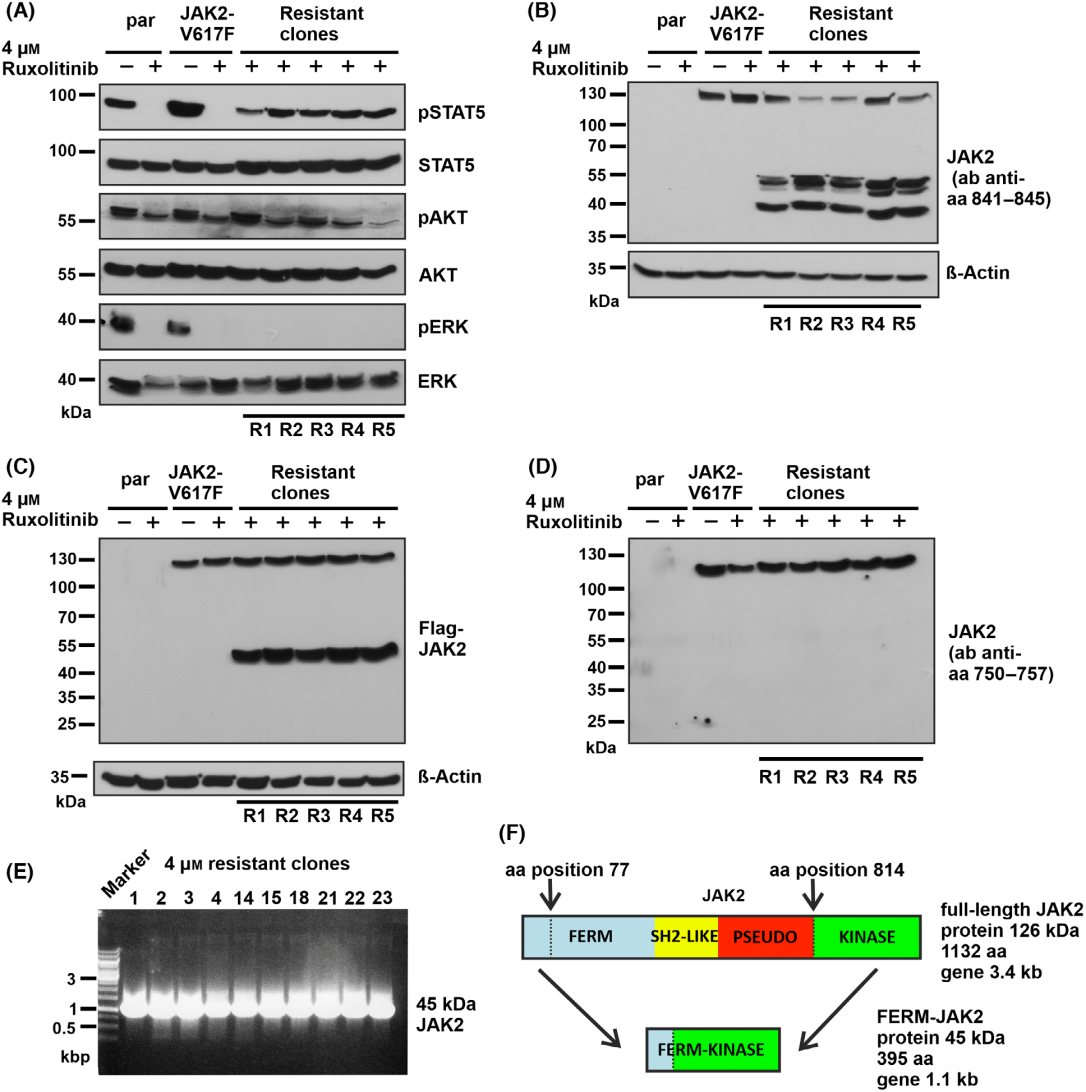
Ruxolitinib‐resistant Ba/F3 cell clones harbor a truncated JAK2 variant. (A) Immunoblot analysis of STAT5, AKT, and ERK signaling in 4 μm ruxolitinib‐resistant Ba/F3 cell clones R1–R5 (*n* = 5 different resistant clones). Par‐parental Ba/F3 cells. (B) Immunoblot analysis of resistant clones using a JAK2 antibody recognizing amino acids (aa) 841–845 shows presence of a 45 kDA form (*n* = 5 different resistant clones). (C) Immunoblot analysis of resistant clones using a FLAG antibody detecting the n‐terminal sequence next to JAK2‐V617F cDNA (*n* = 5 different resistant clones). (D) An antibody raised against aa 750–757 fails to identify the 45 kDa JAK2 variant in drug‐resistant clones in an immunoblot (*n* = 5 different resistant clones). A representative image of *n* = 2 two independent experiments is shown (A, B, C and D). (E) PCR analysis of the JAK2 cDNA isolated from 4 μm ruxolitinib‐resistant clones (*n* = 10 different resistant clones). (F) Schematic representation of sequencing results that revealed an in‐frame deletion of aa 77 to aa 814.

Ruxolitinib is a potent JAK1/JAK2 specific inhibitor that exhibits remarkable clinical activity in JAK2‐V617F mediated MPNs [25]. Unlike most tyrosine kinase inhibitor (TKI)‐treated hematological malignancies, no drug‐resistant mutations have been reported so far in ruxolitinib‐treated MPNs. To identify possible mechanisms of resistance, we used a screening strategy based on the Ba/F3 cell line transformed by JAK2‐V617F. After continuous exposure to ruxolitinib for 14 to 20 days, a high number of resistant clones emerged in 1000 nm (65/2 × 10^7^ cells seeded) and 2000 nm (42/2 × 10^7^ cells seeded) concentrations, whereas the yield decreased for cell treated with 4000 nm (26/2 × 10^7^ cells), suggesting that high ruxolitinib concentration prevents the generation of drug‐resistant clones. Sequencing of ruxolitinib‐resistant clones from all concentrations did not display any point mutation either in kinase or pseudokinase domain of JAK2 (Table S1). Western blot analysis of 4000 nm drug‐resistant clones results showed persistent activation of STAT5 in all clones analyzed (R1, R2, R3, R4, and R5) and activation of AKT only in R1 and R3, indicating clonal heterogeneity. None of the drug‐resistant clones displayed persistent activation of ERK1/2 (Fig. 1A). Surprisingly, analysis of JAK2 protein levels using an antibody detecting the c‐terminal amino acids 841–847 revealed a previously unknown protein band at a molecular weight of 45 kDa in addition to full‐length (130 kDa) JAK2 protein (Fig. 1B). Using an antibody recognizing the n‐terminal FLAG‐tag, we could also identify this smaller protein in drug‐resistant clones but not in drug‐sensitive clones (Fig. 1C). An antibody raised against the JAK2 pseudokinase domain (amino acids 751–757) of JAK2 did not detect the 45 kDa JAK2 variant in drug‐resistant clones (Fig. 1D). These results point toward presence of both n‐terminus and c‐terminus with deletion of the pseudokinase domain in the truncated protein. To identify the full sequence of the 45 kDa JAK2 variant, we designed forward primers recognizing the FLAG DNA sequence and reverse primer binding the DNA sequence corresponding to the C‐terminal antibody recognition site. This strategy yielded a 300 bp fragment in drug‐resistant clones whereas 2500 bp sequence in drug sensitive clones (Fig. S1A). Finally, using the FLAG DNA sequence as forward primer and kinase domain c‐ter sequence as reverse primer, we could amplify the whole sequence of the 45 kDa JAK2 variant which is approximately 1150 bp (Fig. S1B and Fig. 1E). Sequencing of the PCR product showed that a major part of FERM domain, as well as the whole SH2‐like domain and pseudokinase domain are completely lost, leaving an in‐frame fusion protein consisting of the n‐terminal 77 amino acids together with residues 814–1132 amino acids of the kinase domain. We named this variant FERM‐JAK2 (Fig. 1F). Analysis of all the drug‐resistant clones results showed that 80% of the clones acquired the 45 kDa JAK2 variant (Table S2).

### 3.2 FERM‐JAK2 has transforming capacity and activates STAT5 via a non‐canonical pathway

**Fig. 2:**
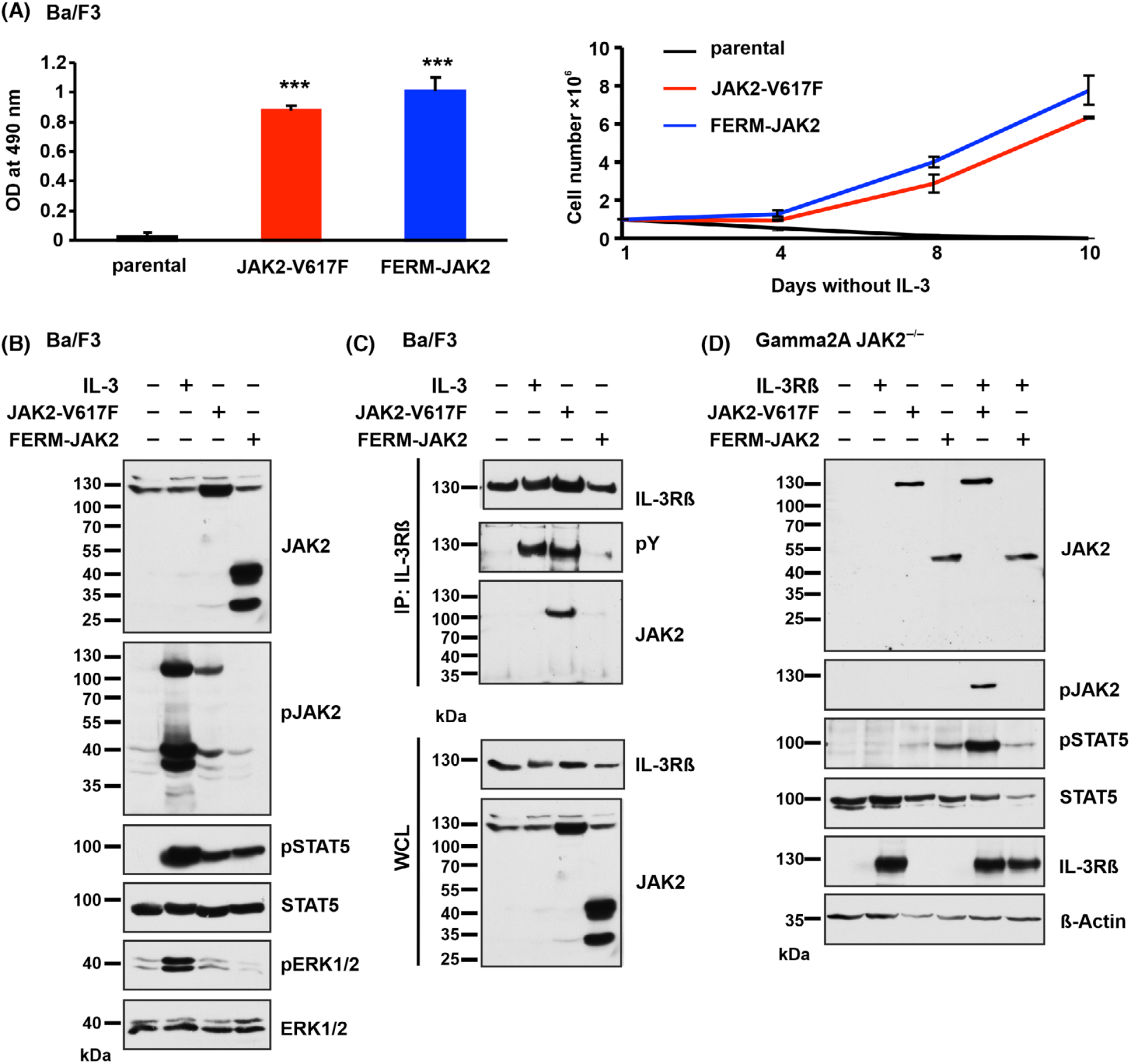
FERM‐JAK2 transforms Ba/F3 cells and activates STAT5 via a non‐canonical pathway. (A) Left panel: Proliferation of parental Ba/F3 cells and Ba/F3 cells expressing FERM‐JAK2 or JAK2‐V617F in the absence of IL‐3 was quantified by the relative optical density (OD) after 96 h using an MTS (3‐(4,5‐dimethylthiazol‐2‐yl)‐2,5‐diphenyltetrazolium bromide)‐based assay. Right panel: Absolute cell numbers over time were measured in the absence of IL‐3 by trypan blue exclusion (*n* = 3). ***P < 0.001 compared to parental cells by Student's *t* test. Data are shown as mean ± standard deviation (SD). (B) Immunoblot analysis of serum‐starved Ba/F3 cells expressing FERM‐JAK2 or JAK2‐V617F. A representative image of *n* = 2 two independent experiments is shown. (C) IL‐3Rβ immunoprecipitation (IP) analysis of Ba/F3 cells expressing FERM‐JAK2 or JAK2‐V617F. pY, phosphotyrosine; WCL, whole cell lysate. A representative image of *n* = 3 three independent experiments is shown. (D) Immunoblot analysis of Gamma2A cells stably expressing mock vector, FERM‐JAK2 or JAK2‐V617F in combination with or without IL‐3Rβ chain. A representative image of *n* = 3 three independent experiments is shown.

To test whether FERM‐JAK2 retains transforming capacity, we stably introduced FERM‐JAK2 into IL‐3‐dependent murine Ba/F3 cells. FERM‐JAK2 led to strong cytokine independent growth to the Ba/F3 cells as determined by both MTS assay and cell counting, indicating that FERM‐JAK2 acts as an oncogene. As described previously, JAK2‐V617F led to factor independent growth (Fig. 2A) [4, 22]. When we focused on the phosphorylation status, we found that FERM‐JAK2, in contrast to JAK2‐V617F, lacks constitutive kinase autophosphorylation of tyrosine residues 1007 and 1008. However, strong activation of STAT5, but not ERK could be detected (Fig. 2B), suggesting that FERM‐JAK2 is able to activate STAT5 without constitutive autophosphorylation in a ligand independent manner.

Canonical STAT5 activation involves phosphorylation of cytokine receptors after binding to JAK2. In order to investigate FERM‐JAK2 signaling in more detail, we analyzed IL‐3 receptor beta (IL‐3Rβ), which is constitutively phosphorylated by JAK2‐V617F. In line with the lack of autophosphorylation, FERM‐JAK2 also fails to associate with the IL‐3Rβ chain (Fig. 2C). To further confirm the notion that FERM‐JAK2 activates STAT5 without receptor binding, we ectopically expressed FERM‐JAK2 or JAK2‐V617F in Gamma2A cells, which lack intrinsic JAK2 and IL‐3Rβ. Strikingly, FERM‐JAK2 displayed strong activation of STAT5 in these cells and IL‐3Rβ reconstitution did not further enhance STAT5 phosphorylation, indicating that FERM‐JAK2 activates STAT5 independent of interaction with cytokine receptor as shown before [26]. JAK2‐V617F alone did not display any activity in Gamma2A cells and STAT5 activation was only observed after IL‐3Rβ reconstitution, as shown previously [22].

In order to further validate our finding, we also reconstituted the Ba/F3‐FERM‐JAK2 and Ba/F3‐JAK2‐V617F cells with hemagglutinin‐tagged erythropoietin receptor (HA‐EpoR), since EpoR is the most physiological receptor for the JAK2 activation. We found that FERM‐JAK2 transforms the EpoR‐Ba/F3 cells as efficiently as JAK2‐V617F (Fig. S2A). Similar to IL‐3Rβ, immunoprecipitation (IP) and Epo stimulation results suggest that FERM‐JAK2 fails to activate and associate with the EpoR (Fig. S2B,C).

### 3.3 FERM‐JAK2 binds STAT5 directly by constitutive dimerization

**Fig. 3:**
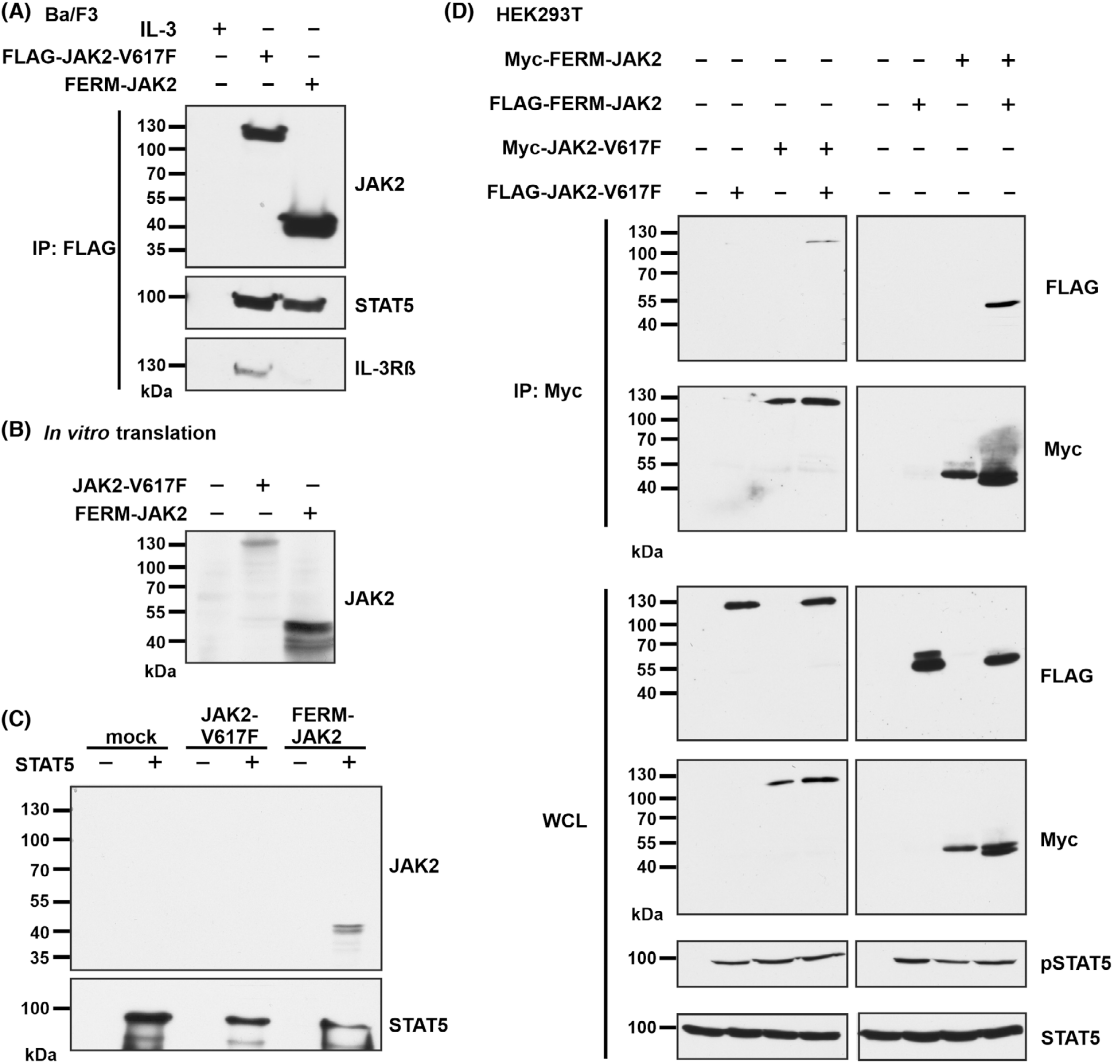
FERM‐JAK2 directly binds STAT5 via constitutive dimerization. (A) FLAG immunoprecipitation (IP) analysis of Ba/F3 cells expressing FLAG‐FERM‐JAK2 or FLAG‐JAK2‐V617F. A representative image of *n* = 3 three independent experiments is shown. (B) Immunoblot analysis of *in vitro* translated FERM‐JAK2 or JAK2‐V617F. A representative image of *n* = 3 three independent experiments is shown. (C) Immunoblot analysis of *in vitro* translated FERM‐JAK2 or JAK2‐V617F incubated with purified STAT5 after washing. A representative image of *n* = 3 three independent experiments is shown. (D) Myc IP analysis of HEK‐293T cells co‐expressing FLAG‐tagged and Myc‐tagged FERM‐JAK2 or JAK2‐V617F. WCL, whole cell lysate. A representative image of *n* = 3 three independent experiments is shown.

As FERM‐JAK2 activates STAT5 without cytokine receptor association, we suspected that FERM‐JAK2 directly binds STAT5. Subcellular fractionation demonstrated that FERM‐JAK2 is strictly localized in cytoplasm whereas JAK2‐V617F additionally exists in a small fraction in the nucleus (Fig. S3), confirming previous studies [27]. Using JAK2 co‐IP, we indeed found evidence that FERM‐JAK2 directly associates with STAT5 without receptor involvement, whereas the interaction of JAK2‐V617F with STAT5 is mediated by IL‐3Rβ (Fig. 3A). To further confirm the direct association of STAT5 to FERM‐JAK2, we used *in vitro* translation of both FERM‐JAK2 and JAK2‐V617F (Fig. 3B) and performed binding studies with purified STAT5 protein. In line with the co‐IP results, FERM‐JAK2, but not JAK2‐V617F, directly interacts with STAT5 *in vitro* (Fig. 3C). In order to analyze whether the direct FERM‐JAK2‐STAT5 association is due to constitutive kinase dimerization, we inserted FERM‐JAK2 and JAK2‐V617F in both FLAG‐tagged and Myc‐tagged expression constructs. All FLAG‐ or Myc‐tagged proteins showed equal level of STAT5 activation. Strikingly, IP of Myc‐tagged FERM‐JAK2 resulted in increased co‐IP of FLAG‐tagged FERM‐JAK2. In contrast, IP of Myc‐tagged JAK2‐V617F resulted in the co‐IP of FLAG‐tagged JAK2‐V617F (Fig. 3D), as previously described [22]. These results suggest that FERM‐JAK2 molecules dimerize constitutively, resulting in direct constitutive STAT5 interaction and phosphorylation.

### 3.4 FERM‐JAK2 is highly resistant to ATP‐competitive inhibitors

**Fig. 4:**
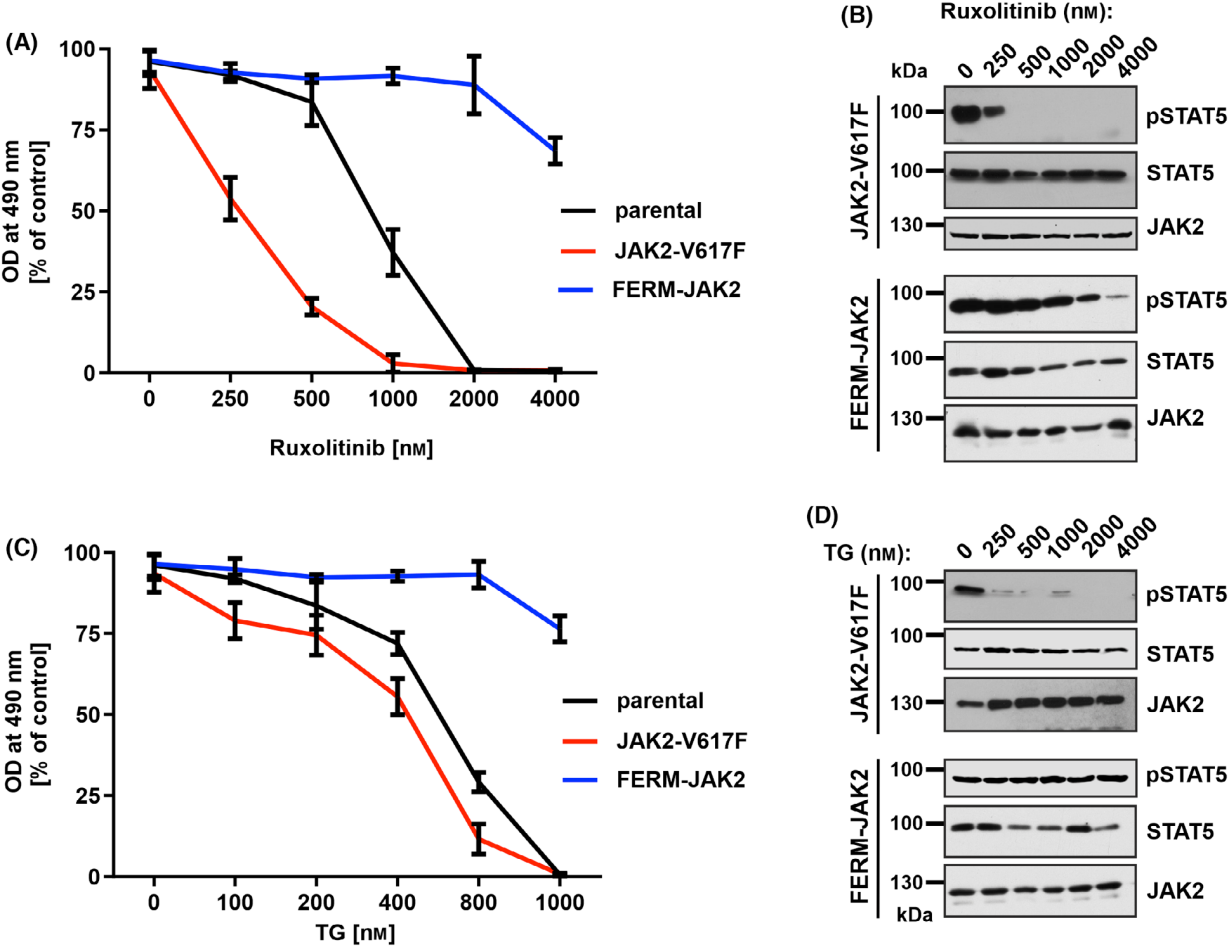
FERM‐JAK2 confers resistance to JAK2‐ATP competitive inhibitors. (A) MTS (3‐(4,5‐dimethylthiazol‐2‐yl)‐2,5‐diphenyltetrazolium bromide)‐based cell proliferation analysis of Ba/F3 cells expressing JAK2 mutants cultured with indicated concentration of ruxolitinib for 48 h. OD, optical density. Data are shown as mean ± standard deviation (SD) (*n* = 3). (B) Immunoblot analysis of Ba/F3 cells expressing JAK2 mutants cultured with indicated concentration of ruxolitinib for 48 h. A representative image of *n* = 2 two independent experiments is shown. (C) MTS‐based cell proliferation analysis of Ba/F3 cells expressing JAK2 mutants cultured with indicated concentration of TG101348 (TG) for 48 h. Data are shown as mean ± standard deviation (SD) (*n* = 3). OD, optical density. (D) Immunoblot analysis of Ba/F3 cells expressing JAK2 mutants cultured with indicated concentration of TG101348 (TG) for 48 h. A representative image of *n* = 2 two independent experiments is shown.

Since we identified FERM‐JAK2 in ruxolitinib‐resistant clones, we decided to test whether indeed FERM‐JAK2 is able to confer drug resistance to the cells. Hence, we took stably transfected Ba/F3 cells expressing FERM‐JAK2 or JAK2‐V617F and cultivated them in presence of increasing concentrations of ruxolitinib. We found that Ba/F3‐FERM‐JAK2 cells are highly resistant to ruxolitinib up to concentrations of 4 μm, whereas JAK2‐V617F‐positive cells are sensitive to ruxolitinib as demonstrated previously [28]. Parental Ba/F3 cells are less sensitive to ruxolitinib compared to Ba/F3‐JAK2‐V617F cells (Fig. 4A). FERM‐JAK2 expressing cells showed persistent STAT5 activation at 2 μm ruxolitinib concentration, which is slightly reduced at 4 μm (Fig. 4B). These data provide evidence that indeed FERM‐JAK2 confers resistance to ruxolitinib.

We then investigated cross‐resistance to fedratinib (TG101348), another potent JAK2 inhibitor with high activity in JAK2‐V617F, JAK2 exon 12 mutation and MPL‐W515K positive patients [29]. Similar to ruxolitinib, treatment of Ba/F3‐FERM‐JAK2 cells with fedratinib does not impair cell growth or STAT5 activation, even at high (4 μm) concentrations. In contrast, Ba/F3‐JAK2‐V617F cells are highly sensitive toward fedratinib (Fig. 4C,D) confirming previous studies [30].

In order to overcome the resistance of FERM‐JAK2 cells to ATP‐competitive inhibitors, we focused on heat shock protein (HSP) 90 inhibitors. JAK2‐V617F is a client protein of HSP90, and cells harboring JAK2 kinase inhibitor resistant mutations have been demonstrated to be sensitive to HSP90 inhibitors [31]. When we treated Ba/F3‐FERM‐JAK2 cells with the HSP90 inhibitors 17‐AAG and geldanamycin, both substances exhibit strong inhibition of cell proliferation as well as downregulation of the FERM‐JAK2 protein (Fig. S4A–C). These results indicate that FERM‐JAK2 is highly dependent on the HSP90 pathway for its proper folding and HSP90 inhibitors could be used as therapeutic agents against JAK2 TKI resistant variants.

### 3.5 Activation loop tyrosine phosphorylation is dispensable for FERM‐JAK2 activation

**Fig. 5:**
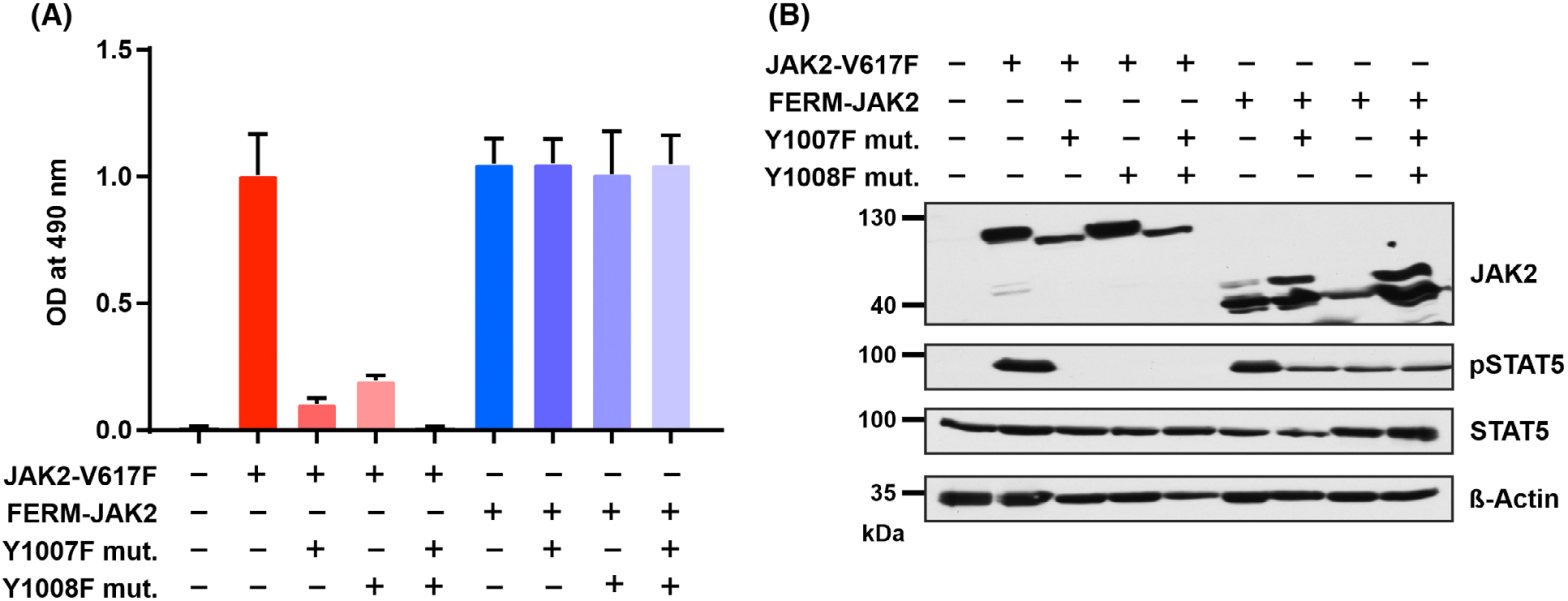
Activation loop phosphorylation is dispensable for FERM‐JAK2 activation. (A) MTS (3‐(4,5‐dimethylthiazol‐2‐yl)‐2,5‐diphenyltetrazolium bromide)‐based cell proliferation analysis of Ba/F3 cells expressing FERM‐JAK2 or JAK2‐V617F plus activation loop mutations Y1007F and/or Y1008F. A representative result (*n* = 2) from three independent experiments is shown. Data are shown as mean ± standard deviation (SD). OD, optical density. (B) Immunoblot analysis of serum starved Ba/F3 cells expressing FERM‐JAK2 or JAK2‐V617F phospho‐deficient mutants Y1007F and/or Y1008F. A representative image of *n* = 2 two independent experiments is shown.

We next set out to elucidate the molecular mechanism of inhibitor resistance conferred by FERM‐JAK2. In general, ATP‐competitive inhibitors block the ATP‐binding pocket within the kinase domain. Consequently, resistance primarily occurs through structural alterations that deny inhibitor access to the pocket. FERM‐JAK2 however, does not display any point mutations in the ATP‐binding pocket, therefore we focused on the activation loop, which regulates kinase activity and accessibility. JAK2 is a structurally very plastic enzyme which can be present in an active or inactive conformation. The switch to an active kinase state can be triggered by phosphorylation of tandem tyrosines located within the activation loop, moving the loop away and thereby unblocking the active site. X‐ray crystallographic studies of the isolated JAK2 kinase domain demonstrated that ruxolitinib and several other JAK2 inhibitors bind the ATP pocket when the activation loop tyrosines are phosphorylated [32]. To study the impact of the activation loop in the FERM‐JAK2 background, we substituted either the tyrosine residue Y1007 or Y1008, or both with phenylalanine. However, none of these variants prevent FERM‐JAK2 from activating STAT5 and transforming Ba/F3 cells, whereas in contrast, they all lead to complete inactivation of JAK2‐V617F (Fig. 5A,B). These results suggest that activation loop tyrosines Y1007 and Y1008 play a dispensable role in FERM‐JAK2 activation, contrary to JAK2‐V617F. However, more detailed structural and functional studies are required to explain the resistant phenotype of FERM‐JAK2 against type I JAK2 inhibitors.

We then mutated all JAK2 kinase domain tyrosines to phenylalanine in both FERM‐JAK2 and JAK2‐V617F to determine tyrosine residues necessary for FERM‐JAK2 activation. We found that tyrosines Y868, Y913 + Y918, and Y966 are solely required by FERM‐JAK2 to activate STAT5, compared to JAK2‐V617F depending on Y934 + Y940 in addition to Y1007 and Y1008. Interestingly, only Y972 is a tyrosine crucial for autophosphorylation of both FERM‐JAK2 and JAK2‐V617F (Fig. S5A–D). However, it is noteworthy that most tyrosines in JAK2 are essential for structural integrity, like Y972 which most likely forms a hydrogen bonding to S904 [33]. Therefore, the phenylalanine mutants we created here might have affect not just JAK phosphorylation. Thus, in‐depth structural and functional analyses are required to conclude the mode of FERM‐JAK2 activation.

### 3.6 FERM‐JAK2 induces an accelerated MPN phenotype in the murine model

**Fig. 6:**
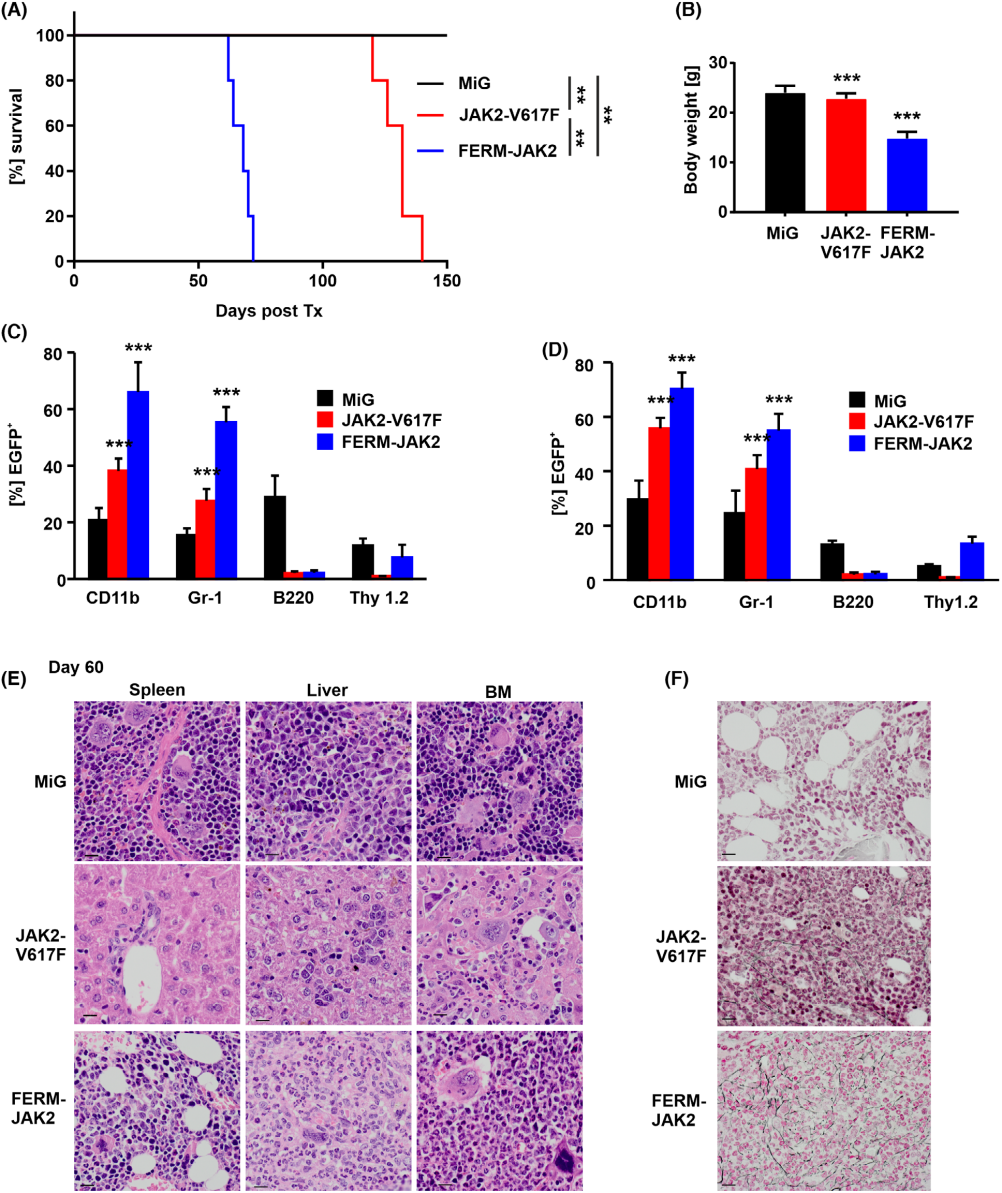
FERM‐JAK2^+^ mice succumb to an accelerated MPN with myelofibrosis. Two independent experiments were analyzed, including a total of *n* = 17 mice receiving bone marrow (BM) transduced with FERM‐JAK2, *n* = 18 JAK2‐V617F^+^ bone marrow (BM) and *n* = 10 empty vector control bone marrow (BM) cells. (A) Kaplan–Meier survival plot of recipient mice, FERM‐JAK2 mice display accelerated disease (*n* = 5 for all groups). **P < 0.001 by Logrank test. Tx, transplantation. (B) FERM‐JAK2 mice show a significant decrease of total body weight 60 days after transplantation compared to MiG (MSCV‐ires‐GFP) empty vector control mice (*n* = 5). ***P < 0.001, n.s., not significant, both compared to MiG (MSCV‐ires‐GFP) by Student's *t* test. Data are shown as mean ± standard deviation (SD). (C, D) Flow cytometric analysis of (C) splenocytes and (D) BM cells taken 60 days after transplantation. Values represent Mean ± SEM of the transplanted animals. ***P < 0.001 compared to MiG by Student's *t* test. (E) Histopathologic analysis (hematoxylin and eosin staining, ×400) revealed hyperplastic, left‐shifted myelopoiesis granulopoiesis, erythropoiesis, and moderate increased megakaryopoiesis. Scale bar represents 15 μm. Notably, infiltrates are very dense in FERM‐JAK2 mice compared to JAK2‐V617F mice. (F) Hematoxylin/eosin and reticulin staining of representative tissue samples 60 days after transplantation (400×). BM obtained from FERM‐JAK2 mice shows left‐shifted increase of myeloid cells and a marked presence of collagen fibers, similar to the increase of reticulin fibers in human myeloproliferative disorders. Slides were viewed with a Zeiss Axioplan 2 microscope (Göttingen, Germany) (40×/0.75 NA Plan‐Neofluar air objective). Scale bar represents 15 μm. Images were acquired using a Zeiss Axiocam MRc 5 camera and were processed with axiovision rel 4.6 scanning software (CarlZeissMicroscopy GmBH, Jena, Germany).

In order to investigate whether FERM‐JAK2 is able to induce a disease in the murine model, we transplanted lethally irradiated Balb/c mice with bone marrow (BM) cells harboring either FERM‐JAK2, JAK2‐V617F, or empty vector in two independent experiments. Strikingly, recipient mice succumbed to FERM‐JAK2‐induced disease with a median survival of 68 days, together with reduction of the body weight. In contrast, JAK2‐V617F transplanted mice died after a median 132 days (Fig. 6A,B). BM and spleen organ analysis results revealed a granulocytic disease for both FERM‐JAK2 and JAK2‐V617F transplanted mice with an increase in CD11b and Gr‐1 positive cells in (Fig. 6C,D). Microscopically, spleens derived from FERM‐JAK2^+^ and JAK2‐V617F^+^ mice showed a marked leukemic infiltration with a left shifted granulopoiesis, erythropoiesis, and moderate increased megakaryopoiesis. Notably, infiltrates are denser in FERM‐JAK2 mice compared to JAK2‐V617F mice (Fig. 6E). Moreover, FERM‐JAK2 transplanted mice showed grade II myelofibrosis already 60 days after transplantation, whereas JAK2‐V617F transplanted mice did not display any signs of myelofibrosis at that stage (Fig. 6F). FERM‐JAK2^+^ recipient mice developed signs of MPN similar to JAK2‐V617F‐induced disease with increased WBC, hematocrit, hemoglobin and granulocytes in peripheral blood (Fig. S6A,B). In addition to elevated blood counts, FERM‐JAK2 also induced a profound splenomegaly with a median spleen weight of 476 mg, compared to 292 mg for JAK2‐V617F^+^ and 113 mg for empty vector control mice (Fig. S6C).

## 4 Discussion

BCR‐ABL negative MPNs such as PV, ET and PMF are frequently associated with the V617F mutation in JAK2 [3, 4, 5, 7, 13]. In addition to MPNs, constitutive JAK2 signaling is also involved in several solid tumors and other lymphoid malignancies [34, 35, 36]. Small molecule inhibitors such as ruxolitinib, TG101348 and CEP‐701 have shown remarkable clinical activity in clinical trials of PMF patients [17, 37]. In CML and GIST, it has been demonstrated that acquired resistance to the ABL kinase inhibitor imatinib is due to the emergence of secondary kinase domain mutations. More than 70 different exchanges have been described that confer drug resistance in the CML [38]. However, no inhibitor resistant variant has been described in MPN treated with JAK inhibitors so far. In this study, we identified a 45‐kDa novel JAK2 variant, which drives resistance to available JAK2 inhibitors.

In our cell‐based screening approach, the yield of resistant clones was high at low ruxolitinib concentrations and decreased with higher ruxolitinib concentration. Cell clones resistant to low ruxolitinib concentrations did not display any JAK2 point mutations neither in the kinase nor in the pseudokinase domain. A possible explanation may be that amino acids variations which diminish inhibitor binding also compromise JAK2 kinase activity. In line with these findings, Koppikar et al. [39] showed that occurrence of genetic resistance by point mutations in JAK2 is rare and that heterodimeric JAK–STAT activation is one mechanism for MPN cell to persist under therapy. Another study identified few novel JAK2 inhibitor resistant variants using random mutagenesis, which only induced a modest increase in half‐maximum inhibitory concentration (IC50) [28]. Weigert et al. [31] identified three novel JAK2 variants, G935R, Y931C and E864K, conferring resistance against the inhibitor BVB808. These studies provide evidence that the low frequency of JAK2 inhibitor resistance mutations might be related to the limited repertoire of kinase domain residues that could possibly mediate that resistance.

Our screen recovered the FERM‐JAK2 variant as the most frequent event (80%) capable of supporting cell growth in the presence of ruxolitinib. So far, no other study has detected this 45 kDa protein, which may be due to methodological differences. The studies mentioned above carried out random mutagenesis before applying selection pressure, which might favor point mutations over truncation variants. In contrast, we decided to avoid any mutagenesis steps, which represents the clinical situation much more closely. An alternative explanation could be that other studies relied on detection antibodies binding to the deleted protein part and therefore missed the short form of JAK2. In addition, our results also provide evidence that sequencing solely the kinase domain might not be sufficient to identify all relevant TKI escapes mechanisms. This finding warrants the importance of whole exome next‐generation sequencing (NGS) including splice variants for ruxolitinib‐resistant MPN patients.

Our results are in line with findings in BRAF‐V600E positive melanoma patients, where resistance against the TKI vemurafenib is mediated by the generation of a truncated BRAF splice variant (p61BRAF[V600E]). This protein is generated by deletion of exons 4 to 8 including critical domains for RAF activation, most notably the RAS‐binding domain (RBD) and the cysteine–rich domain (CRD). It was shown that p61BRAF(V600E) mediates enhanced dimerization compared to full‐length BRAF protein [40]. In contrast, the 45 kDa JAK2 variant observed here is not the result of a splice event, as there are no known splice sites at the protein fusion points. Moreover, the fusion event took place within the JAK2‐V617F cDNA introduced into the Ba/F3 cell line for transformation, as indicated by the sequencing strategy utilizing forward primers targeting the 5′ FLAG tag. In mechanistic studies, we demonstrated that FERM‐JAK2 constitutively activates STAT5 via a non‐canonical pathway without receptor interaction. These results support a previous observation that receptor unbound FERM domain interacts with the JH1/JH2 domains, thereby preventing inappropriate activity of the kinase. Recruitment of JAK2 to the receptor complex leads to release of inhibitory constraints exerted by the FERM domain on the JH1/JH2 domains [41]. Based on these data, we assume that FERM‐JAK2 is constitutively active due to lack of around 80% of the FERM domain, rendering it unable to restrict kinase activity. In addition, FERM‐JAK2 is also characterized by deletion of pseudokinase domain, which is a known negative regulator of the kinase domain [42, 43, 44]. Moreover, JAK2 protein levels are negatively regulated by SOCS proteins such as SOCS‐1 and SOCS‐3, which are recruit to JAK kinases via phosphorylated activation loop tyrosines Y1007 and Y1008 [45]. Here we found that these two crucial tyrosines are not phosphorylated in Ba/F3‐FERM‐JAK2 cells, which might contribute to enhanced stabilization of FERM‐JAK2 due to lack of SOCS protein binding (data not shown). Our results also demonstrate that FERM‐JAK2, contrary to full‐length JAK2, dimerizes constitutively similar to p61BRAF(V600E) and this constitutive dimerization leads to drug resistance [40].

Apart from its cell transforming capacity, FERM‐JAK2 is also a potent driver of resistance toward ATP‐competitive inhibitors, which is most likely due to an altered conformation that abrogates drug binding to the catalytically active kinase domain. Similar finding has been made with BCR‐ABL mutations rendering CML cells resistant against imatinib. These variants have been shown to destabilize the auto inhibited, inactive conformation of BCR‐ABL which is preferentially bound by imatinib, and shifts the equilibrium toward the active kinase confirmation [46]. In contrast, most JAK2 inhibitors interact with JAK2‐V617F in the active kinase conformation [32] and phosphorylation of activation loop tyrosines acts as a molecular switch between active or in inactive states [47]. Our results demonstrate that activation loop phosphorylation is dispensable for FERM‐JAK2 activation and cellular transformation, suggesting a shift of the equilibrium toward to inactive state of the kinase. However, a complete structural analysis of FERM‐JAK2 would be required to determine the precise mechanism of STAT5 activation and inhibitor resistance.

In our study, we have also uncovered a potential therapeutic strategy to overcome resistance mediated by FERM‐JAK2 through inhibition of HSP90. HSP90 acts as a molecular chaperone with major roles in the maturation of client proteins including a multitude of fusion kinases and oncogenic proteins. Thus, HSP90 inhibitors like geldanamycin or zelavespib (PU‐H71) have proven their value in the treatment of myeloma and other cancers [48], as well as in murine models of JAK2‐V617F and MPL driven MPNs. HSP90 inhibition has also been shown to downregulate JAK2 protein levels *in vitro* [49]. Our results further underline the importance of clinical evaluation of HSP90 inhibitors in drug refractory JAK2 driven MPNs.

Notably, FERM‐JAK2 cannot be detected by most Sanger sequencing approaches, which are still in use for the routine inspection of JAK2 point mutations in MPN patients. Even next‐generation sequencing might not reliably detect FERM‐JAK2 if present at low allele frequency. Workflows used for whole exome sequencing (WES) analysis approved for routine clinical diagnostics typically include multiple filtering steps, e.g., only nonsynonymous mutations listed by the Exome Aggregation Consortium or gnomAD with a variant allele frequency of > 5% and a minor allele frequency of < 0.1% are reported [50]. Coverage represents another important factor, rare variants covered by only 1–2 reads might be missed if the coverage is too low (< 100). In order to detect the FERM‐JAK2 variant, either the filtering strategies could be adapted or fusion detection could be improved by implementing molecular barcodes spanning different exon regions of the JAK2. Our results therefore underline the urgent need for the use of WES deep sequencing optimized for detection of JAK2 length variants for clinical monitoring of MPN patients receiving JAK1/2 directed treatments.

Taken together, we have identified a novel constitutively active JAK2 variant which drives resistance to ATP‐competitive inhibitors *in vitro* and induces an accelerated MPN phenotype *in vivo*.

## 5 Conclusion

We found that ruxolitinib resistance is mediated by 45‐kDa novel JAK2 variant (FERM‐JAK2) that exists in constitutively dimerized state and prevents the phosphorylation of activation loop tyrosines. FERM‐JAK2 induces a severe MPN‐like disease in the mouse model. Our results point toward an urgent need for the use of whole exome sequencing in the clinical monitoring of inhibitor‐refractory individuals in order to detect JAK2 variants.

## Conflict of interest

The authors declare no conflict of interest.

## Author contributions

SPG designed the study, SPG, TAM, and CA‐L performed experiments and analyzed data; MR performed and analyzed histological sections; NB and JD provided critical materials, suggested experiments and reviewed the manuscript; SPG and TAM wrote the manuscript.

## Supporting information


**Fig. S1.** Sequencing strategy to identify FERM‐JAK2 in ruxolitinib‐resistant clones.

**Fig. S2.** FERM‐JAK2 transforms EpoR‐Ba/F3 cells and activates STAT5 without EpoR interaction.

**Fig. S3.** FERM‐JAK2 is not present in the nucleus, in contrast to JAK2‐V617F.

**Fig. S4.** FERM‐JAK2 is sensitive to the HSP90 inhibitors 17‐AAG and geldanamycin.

**Fig. S5.** Phosphorylation of FERM‐JAK2 residues Y868, Y913, Y918 and Y972 is crucial for FERM‐JAK2 mediated transformation and STAT5 activation.

**Fig. S6.** FERM‐JAK2 induces an MPN‐like disease in the murine model.

**Table S1.** Ruxolitinib resistance clones did not display mutations in JAK‐family kinases.
**Table S2.** 80% of the 4 μm ruxolitinib resistance clones displayed a 45 kDa JAK2 variant.

## Data Availability

Data sharing not applicable to this article as no datasets were generated or analyzed during the current study.
