# Supplementary figures and images for: A newly identified 45‐kDa JAK2 variant with an altered kinase domain structure represents a novel mode of JAK2 kinase inhibitor resistance
Source: Mol Oncol. 2023 Dec 20;18(2):415–30. doi: 10.1002/1878-0261.13566 (PMC10850816; doi:10.1002/1878-0261.13566)

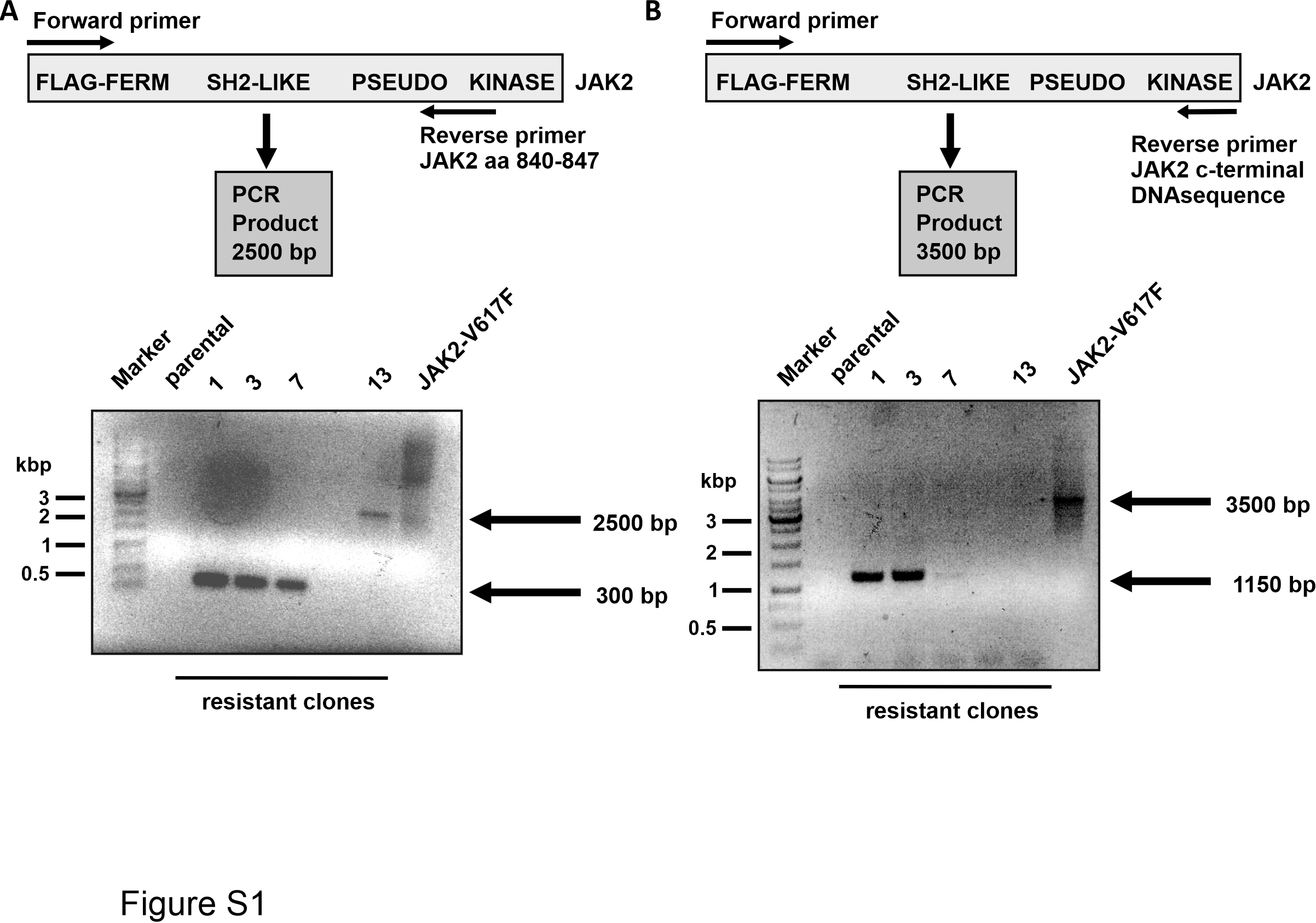

Supplement: Supplementary file 1 — Fig. S1. Sequencing strategy to identify FERM‐JAK2 in ruxolitinib‐resistant clones. [file MOL2-18-415-s004.tiff]

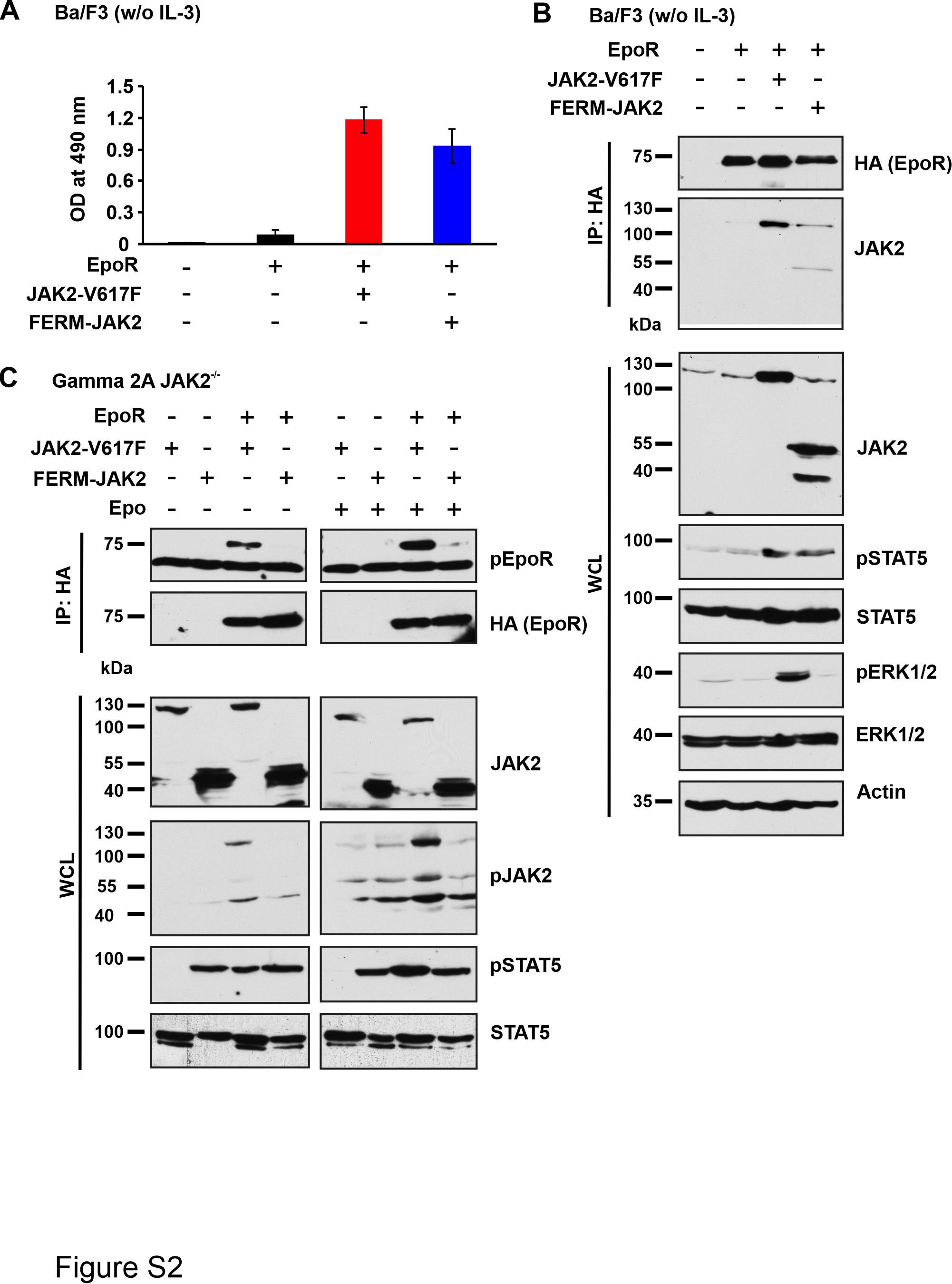

Supplement: Supplementary file 2 — Fig. S2. FERM‐JAK2 transforms EpoR‐Ba/F3 cells and activates STAT5 without EpoR interaction. [file MOL2-18-415-s002.tiff]

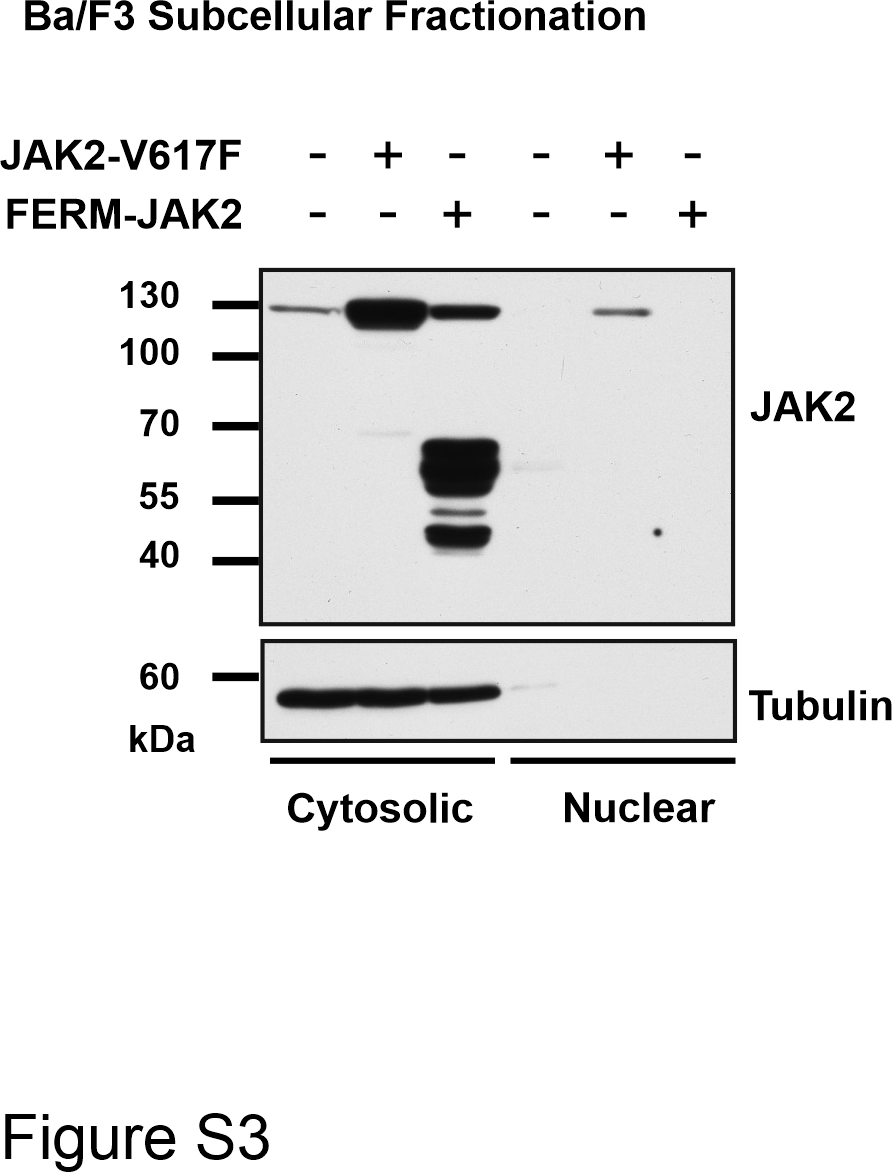

Supplement: Supplementary file 3 — Fig. S3. FERM‐JAK2 is not present in the nucleus, in contrast to JAK2‐V617F. [file MOL2-18-415-s003.tiff]

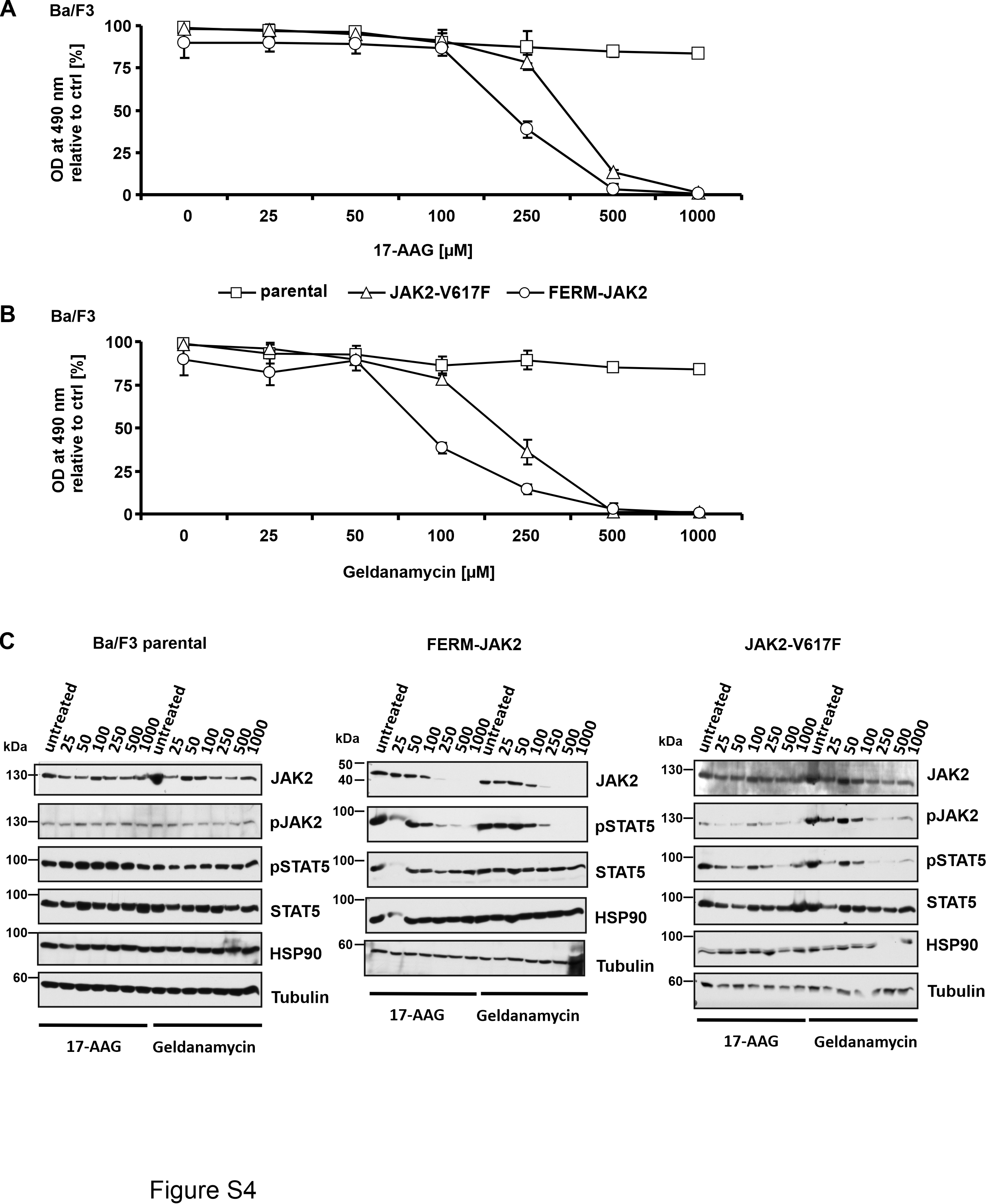

Supplement: Supplementary file 4 — Fig. S4. FERM‐JAK2 is sensitive to the HSP90 inhibitors 17‐AAG and geldanamycin. [file MOL2-18-415-s006.tiff]

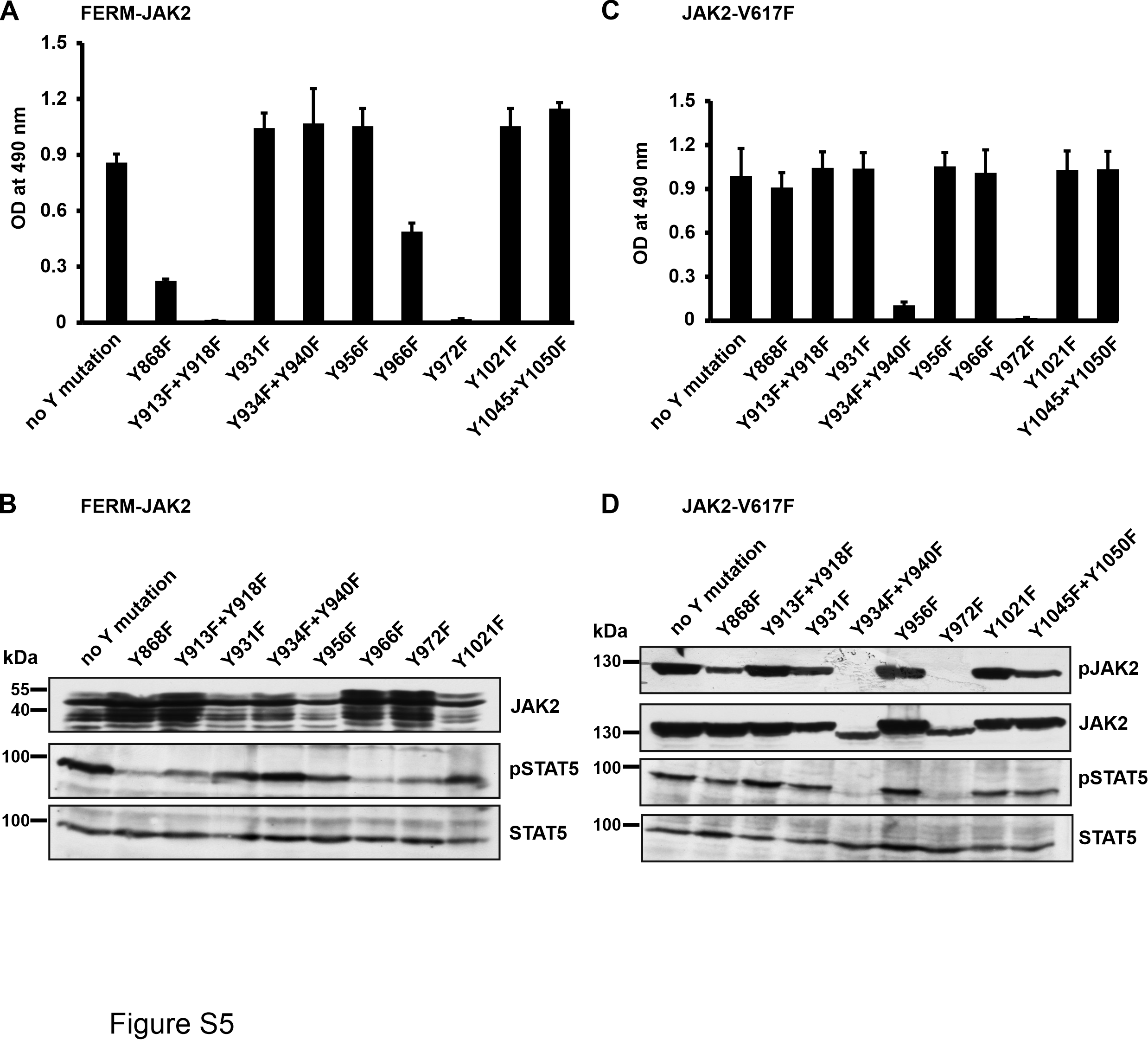

Supplement: Supplementary file 5 — Fig. S5. Phosphorylation of FERM‐JAK2 residues Y868, Y913, Y918 and Y972 is crucial for FERM‐JAK2 mediated transformation and STAT5 activation. [file MOL2-18-415-s005.tiff]

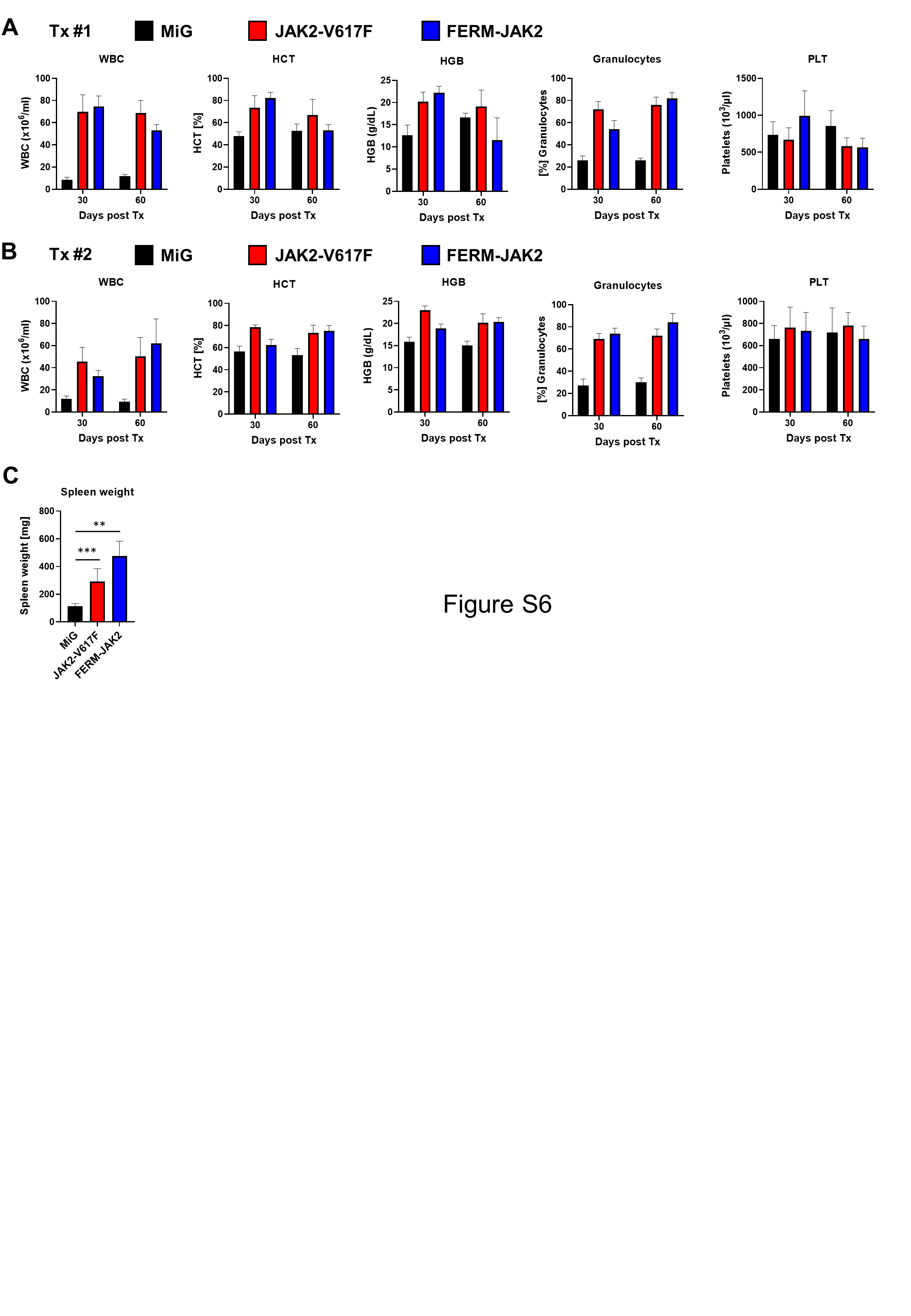

Supplement: Supplementary file 6 — Fig. S6. FERM‐JAK2 induces an MPN‐like disease in the murine model. [file MOL2-18-415-s001.tiff]
