# Supplementary material for: A newly identified 45‐kDa JAK2 variant with an altered kinase domain structure represents a novel mode of JAK2 kinase inhibitor resistance
Source: Mol Oncol. 2023 Dec 20;18(2):415–30. doi: 10.1002/1878-0261.13566 (PMC10850816; doi:10.1002/1878-0261.13566)
Supplement: Supplementary file 7 — Table S1. Ruxolitinib resistance clones did not display mutations in JAK‐family kinases. Table S2. 80% of the 4 μm ruxolitinib resistance clones displayed a 45 kDa JAK2 variant. [file MOL2-18-415-s007.zip › mol213566-sup-0007-Tables.pdf]

Supplementary Table I. Sequencing of ruxolitinib resistant clones

Concentration of ruxolitinib [nM]	Number of resistant clones	Number of clones sequenced	Mutational status
1000	125	65	WT
2000	65	42	WT
4000	26	26	WT

Supplementary Table 2. Sequencing of 4uM ruxolitinib resistant clones displayed 80% of cases 45-kDa JAK2 variant

Concentration of ruxolitinib [nM]	Number of resistant clones	Number of WT clones	Number of clones displayed 45-kDa JAK2
4000	26	5	21
