# Supplementary material for: A newly identified 45‐kDa JAK2 variant with an altered kinase domain structure represents a novel mode of JAK2 kinase inhibitor resistance
Source: Mol Oncol. 2023 Dec 20;18(2):415–30. doi: 10.1002/1878-0261.13566 (PMC10850816; doi:10.1002/1878-0261.13566)
Supplement: Supplementary file 7 — Table S1. Ruxolitinib resistance clones did not display mutations in JAK‐family kinases. Table S2. 80% of the 4 μm ruxolitinib resistance clones displayed a 45 kDa JAK2 variant. [file MOL2-18-415-s007.zip › Updated_Supplement file for FERM-JAK2 manuscript_Molecular Oncology.docx]

**A newly identified 45-kDa JAK2 variant with an altered kinase domain structure represents a novel mode of JAK2 kinase inhibitor resistance**

- Supplemental table S1. Ruxolitinib resistance clones did not display mutations in JAK-family kinases.
- Supplemental table S2. 80% of the 4uM ruxolitinib resistance clones displayed a 45kDa JAK2 variant.
- Figure S1. Sequencing strategy to identify FERM-JAK2 in ruxolitinib resistant clones.
- Figure S2. FERM-JAK2 transforms EpoR-Ba/F3 cells and activates STAT5 without EpoR interaction.
- Figure S3: FERM-JAK2 is not present in the nucleus, in contrast to JAK2-V617F.
- Figure S4. FERM-JAK2 is sensitive to the HSP90 inhibitors 17-AAG and geldanamycin.
- Figure S5: Phosphorylation of FERM-JAK2 residues Y868, Y913, Y918 and Y972 is crucial for FERM-JAK2 mediated transformation and STAT5 activation.
- Figure S6: FERM-JAK2 induces an MPN-like disease in the murine model.

**Supplemental figure legends**

**Table 1: Ruxolitinib resistant clones did not display any mutations in JAK-family kinases**

**Table 2: Sequencing of 4uM ruxolitinib resistant clones displayed 80% of cases 45-kDa JAK2 variant**

**Figure S1. Sequencing strategy to identify FERM-JAK2 in ruxolitinib resistant clones.**

**A** Schematic representation of JAK2 domains and primer target regions in the gene. In an initial PCR approach, a forward primer corresponding to the n-terminal FLAG sequence was designed. The DNA sequence encoding for amino acids 840-847 in the kinase domain was used as a reverse primer. Expected size of PCR product is 2500 base pairs (bp). **B** In a second PCR set, FLAG forward primer was used in combination with a reverse primer targeting the *JAK2* c-terminal sequence. In the drug resistant clones (1, 3 and 7), a 1100 bp sequence corresponding to FERM-JAK2 was amplified, whereas Ba/F3 cells expressing the full length JAK2-V617F display a 3500-nucleotide fragment.

**Figure S2. FERM-JAK2 transforms EpoR-Ba/F3 cells and activates STAT5 without EpoR interaction.**

**A** Proliferation of parental Ba/F3 cells and Ba/F3 cells expressing EpoR, JAK2-V617F or FERM-JAK2 in the absence of IL-3 was quantified by relative optical density (OD) after 96 hours using an MTS-based assay. **B** Immunoblot analysis of serum-starved Ba/F3 cells expressing EpoR, EpoR+JAK2-V617F or EpoR+FERM-JAK2. **C** EpoR immunoprecipitation (IP) analysis of Ba/F3 cells expressing FERM-JAK2 or JAK2-V617F. Whole cell lysate (WCL) shown in the bottom panel with or without Epo stimulation.

**Figure S3: FERM-JAK2 is not present in the nucleus, in contrast to JAK2-V617F.**

Immunoblot of Ba/F3 nuclear and cytoplasmic fractions demonstrates that JAK2-V617F displays mainly cytoplasmic and partial nucleic localization, whereas FERM-JAK2 location is restricted to the cytoplasm.

**Figure S4. FERM-JAK2 is sensitive to the HSP90 inhibitors 17-AAG and geldanamycin.**

**A, B** Ba/F3 cells expressing FERM-JAK2 or JAK2-V617F were treated with increasing concentrations of **A** 17-AAG or **B** geldanamycin and cell proliferation was measured via an MTS-based assay. **C** Parental Ba/F3 cells and Ba/F3 cells expressing FERM-JAK2 or JAK2-V617F were treated with indicated concentrations of 17-AAG and geldanamycin for 3 hours and lysates are subjected to indicated antibodies (C).

**Figure S5: Phosphorylation of FERM-JAK2 residues Y868, Y913, Y918 and Y972 is crucial for FERM-JAK2 mediated transformation and STAT5 activation.**

**A** Phospho-deficient FERM-JAK2 variants harboring exchange of tyrosines (Y) to phenylalanines (F) were transduced into Ba/F3 cells in in order to identify critical phosphorylation sites. Cell growth in absence of IL-3 was determined by relative optical density (OD) after 96 hours using an MTS-based assay. **B** Immunoblot analysis of STAT5 activation in Ba/F3 cells transduced with indicated FERM-JAK2 phospho-deficient variants. **C** Cell growth of Ba/F3 cells harboring phospho-deficient JAK2-V617F variants in absence of IL-3 was determined by relative optical density (OD) after 96 hours using an MTS-based assay. **D** Immunoblot analysis of STAT5 activation in Ba/F3 cells transduced with indicated JAK2-V617F phospho-deficient variants.

**Figure S6: FERM-JAK2 induces an MPN-like disease in the murine model.**

Two independent experiments including **A** (n=5) and **B** (n=10) mice transplanted with BM cells harboring empty vector (MiG), JAK2-V617F or FERM-JAK2 were performed. Peripheral blood was isolated at days 30 and 60 post transplantation and analyzed for white blood cell count (WBC), hematocrit (HCT), hemoglobin (HGB) value, granulocyte percentage and platelet count. **C** Statistical analysis of spleen weight of transplanted mice at day 72 post transplantation (n=5 MiG, n=10 JAK2-V617F, n=5 FERM-JAK2). **p<0.01, ***p<0.001.
